# The Roles of Cruciferae Glucosinolates in Disease and Pest Resistance

**DOI:** 10.3390/plants10061097

**Published:** 2021-05-30

**Authors:** Zeci Liu, Huiping Wang, Jianming Xie, Jian Lv, Guobin Zhang, Linli Hu, Shilei Luo, Lushan Li, Jihua Yu

**Affiliations:** 1 Gansu Provincial Key Laboratory of Aridland Crop Science, Gansu Agricultural University, Lanzhou 730070, China; liuzc@gsau.edu.cn; 2 College of Horticulture, Gansu Agriculture University, Lanzhou 730070, China; wanghp@st.gsau.edu.cn (H.W.); xiejianming@gsau.edu.cn (J.X.); lvjian@gsau.edu.cn (J.L.); zhanggb@gsau.edu.cn (G.Z.); hull@gsau.edu.cn (L.H.); luosl@gsau.edu.cn (S.L.); lils@st.gsau.edu.cn (L.L.); 3 Panzhihua Academy of Agricultural and Forestry Sciences, Panzhihua 617000, China

**Keywords:** *Brassicaceae*, glucosinolates, hydrolytic products, pathogen, insect resistance, secondary metabolites

## Abstract

With the expansion of the area under Cruciferae vegetable cultivation, and an increase in the incidence of natural threats such as pests and diseases globally, Cruciferae vegetable losses caused by pathogens, insects, and pests are on the rise. As one of the key metabolites produced by Cruciferae vegetables, glucosinolate (GLS) is not only an indicator of their quality but also controls infestation by numerous fungi, bacteria, aphids, and worms. Today, the safe and pollution-free production of vegetables is advocated globally, and environmentally friendly pest and disease control strategies, such as biological control, to minimize the adverse impacts of pathogen and insect pest stress on Cruciferae vegetables, have attracted the attention of researchers. This review explores the mechanisms via which GLS acts as a defensive substance, participates in responses to biotic stress, and enhances plant tolerance to the various stress factors. According to the current research status, future research directions are also proposed.

## 1. Introduction

Plants are exposed to complex and highly variable environmental conditions in the course of their growth and development, and are often at risk of death or even extinction under the influence of diverse biotic and abiotic stress factors [1,2,3]. To survive such challenges in their habitats and environments, plants have evolved numerous adaptive mechanisms, including the production of diverse metabolites, which exhibit obvious species specificity [4]. Depending on their structure and type, plant secondary metabolites are mainly divided into terpenoids, phenols, and nitrogen-containing compounds [5,6,7]. Numerous studies have shown that there are about 90,000–200,000 types of metabolites in plants, and they play essential roles in signal transduction, adaptive regulation, growth and development, and plant defense [8,9,10]. Since many of the secondary metabolites act as defenses, it is presumed that biological invasion played a primary role in the evolution of the compounds [11,12].

Among the secondary metabolites, glucosinolates (GLS) are a type of anion hydrophilic secondary metabolite containing nitrogen and sulfur; GLS are water-soluble and can easily be dissolved in ethanol, methanol, and acetone [13,14]. GLS are found in 16 species of dicotyledonous angiosperms, and their contents are relatively high in the Cruciferae, Cleomaceae, and Caricaceae, and especially in the genus *Brassica*, such as in *B. rapa* ssp. *pekinensis*, *B. oleracea*, *B. napus*, *B. juncea*, and *B. rapa*, as well as in *Arabidopsis thaliana* [13,15,16,17,18]. In addition, the GLS biosynthetic pathway has been extensively studied in the model plant *Arabidopsis*, and the regulatory genes have been comprehensively described. Such studies have sparked interest in the unconventional metabolites derived from amino acids, with a lot of research focusing on GLS in *Brassica* plants [19,20,21].

Since the first type of GLS was isolated from mustard seeds, the associated plant species and GLS degradation products have been gradually recognized. Currently, the structures of more than 200 types of GLS have been identified [13,17,18,22], with more than 15 detected in Cruciferae [23]. Naturally occurring GLS have a common chemical structure: the structures are generally composed of β-D-glucosinyl, a sulfide oxime group, and side-chain R groups (including alkyl, hydroxyalkyl, hydroxyalkenyl, alkenyl, methylsulfinylalkyl, methylsulfonylalkyl, methylthioalkyl, arylalkyl, and indolyl) derived from amino acids; furthermore, GLS are generally in the form of potassium or sodium salts [15]. Based on the amino-acid side chain R groups, GLS can be divided into three categories, including aliphatic GLS (side chains are mainly derived from methionine, alanine, valine, leucine, or isopropyl leucine), indole GLS (side chains mainly derived from tryptophan), and aromatic GLS (side chains mainly derived from phenylalanine or tyrosine) [19,24].

GLS are mainly found in plant seeds, roots, stems, and leaf vacuole cells, and are relatively stable in nature with no associated biological activity; conversely, glucosinase (also known as myrosinase), which is responsible for hydrolyzing glucose residues in the GLS core skeleton, is located in specific protein bodies [25,26,27,28]. In intact plants, the hydrolytic systems containing GLS and myrosinase are spatially isolated; however, when tissue is damaged, for example following infestation or mechanical injury, the two rapidly combine, which leads to the rapid formation of GLS hydrolytic products [29,30,31,32,33]. In addition, food processing techniques, such as chopping, juicing, chewing, cooking, high temperature treatment, and thawing, can also break down GLS [34]. The hydrolytic products of GLS breakdown include glucose and unstable sugar glycoside ligands, and the glycoside ligands are rearranged to form isothiocyanates, nitriles, oxazolidinethiones, thiocyanate, epithionitriles, and other products, which all exhibit a wide range of biological activity [35,36,37,38].

GLS and their degradation products influence the taste and flavor of cruciferous vegetables [39,40], and there were significant difference in GSL content among *Brassica* plants; the total GSL content in the freeze-dried samples ranged from 621.15–42,434.21 μmol kg^−1^, with an average value of 14,050.97 μmol kg^−1^ [41]. The spicy taste in radish is caused primarily by volatile allyl, 3-butane, and 4-methyl thiocyanate (ITC). Furthermore, over the past few decades, it was established that some of the metabolite classes containing nitrogen and sulfur exhibit immunosuppressant and anticancer properties [16,35,42,43,44,45]. Sulforaphane, the degradation product of glucoraphanin, exhibits anticancer activity, can relieve neuropathic pain caused by chemotherapy, and has significant inhibitory effects against prostate, rectal, breast, pancreatic, and bladder cancers [46,47,48,49,50,51,52]. In contrast, a progoitrin degradation product, goitrin (5-vinyloxazolidine-2-thione), can cause goiter and abnormalities in the internal organs of animals [53].

In the field, the mustard oil bomb is a major defense mechanism deployed against insect herbivory [54,55,56], pathogen infection [57,58,59,60,61], and various abiotic stress factors (such as drought, low or high temperature, light, and salt stress) [62,63,64,65,66,67,68,69,70]. The findings of such studies have prompted research on the potential application of GLS extracts and metabolites in crop pest and disease control in recent years [8,71,72].

**Figure 1:**
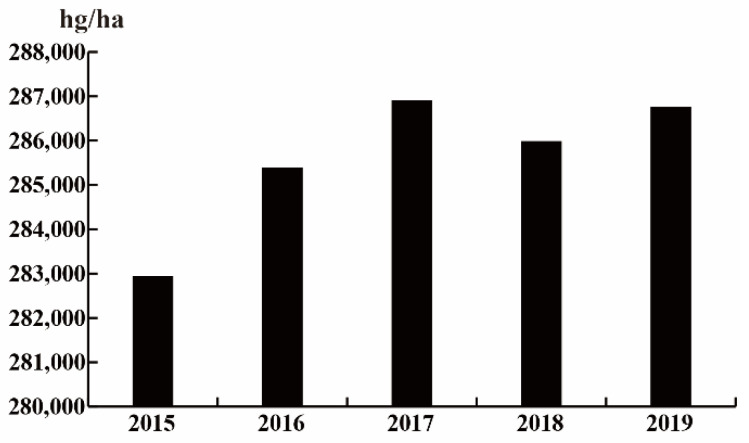
Changes in the cultivated area of cruciferous vegetables in recent years.

Cruciferous vegetables are the largest leafy vegetables in the world, and are widely cultivated globally, and the cultivated area is expanding year by year according to the statistics of Food and Agriculture Organization of the United Nations (FAO) (Figure 1). Cruciferous plants in cultivation are often affected by various fungi (*Plasuwdiophora brassicae*, *Fusarium oxysporum*, *Peronospora parasitica* (Pers), *Sclerotinia sclerotiorum*) [73,74,75,76], bacteria (*Xanthomonas campestris* pv. *campestris, Erwinia carotovora* pv. *carotovora* Dye, *Maculicola pseudomonas syringae*) [77,78,79], and viruses (*Turnip mosaic virus*) [80], which cause club root, Fusarium wilt, downy mildew, sclerotinose, black rot, soft rot, black spot, mosaic, etc. Furthermore, *Plutella xylostella*, aphids, and *Pieris rapae* seriously affect the growth and development of cruciferous plants, and greatly reduce the productivity of cruciferous vegetable farms [81,82,83].

**Figure 2:**
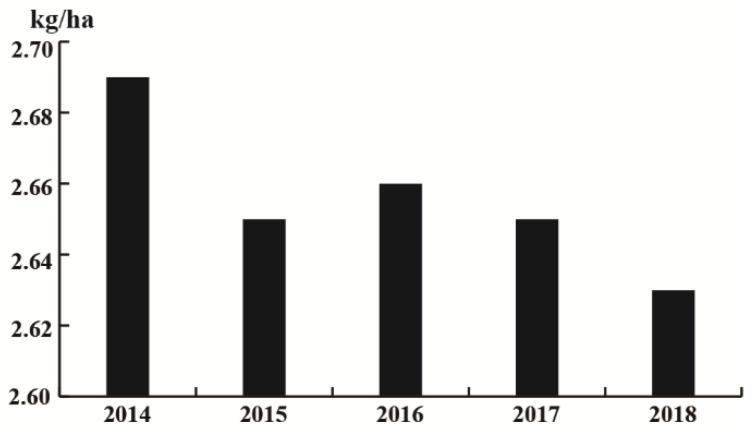
Pesticide application per hectare in recent years.

Currently, chemical control using pesticides is the primary method used to prevent and manage the diseases and insect pests that impair cruciferous vegetable cultivation and productivity. Despite the agricultural production industry currently advocating reducing pesticide application, the use of pesticides is still high according to the data from the FAO (Figure 2). Mass application of chemical pesticides not only increase production costs and deposit excessive pesticide residues on vegetables, but also pose threats to the environment and human health. Consequently, studies and comprehensive data on the potential of GLS derived from Cruciferae to control diseases are required. This review explores and summarizes the latest research on the disease and insect resistance function of GLS, in addition to the underlying resistance mechanisms, in cruciferous plants and in *Arabidopsis*. The present review could provide a theoretical basis for the application of GLS in disease and pest resistance, and the breeding of resistant cruciferous vegetables.

## 2. Defense Response of GLS to Fungal Diseases

The main diseases affecting agricultural production are fungal diseases, which have caused serious losses to the production of cruciferous vegetables. Consequently, investigating the potential effects of GLS extracts and enzymolysis products in resistance against fungal diseases, in addition to their underlying mechanisms of action, could facilitate efforts to improve agricultural productivity in cruciferous crops. Aqueous extracts containing ITC can inhibit the growth of *Alternaria brassicicola* in vitro by 50% [84]. Following exposure to allyl-ITC (Al-ITC), *A. brassicicola* exhibits a response similar to that observed during oxidative stress, based on the results of a study examining the transcriptomic responses of *Arabidopsis* challenged with *A. brassicicola.* In addition, ITCs play major roles in *Arabidopsis* resistance against *Plectosphaerella cucumerina*, *Botrytis cinerea*, *Fusarium oxysporum,* and *Peronospora parasitica* inoculation, demonstrated in a study using a GLS biosynthesis mutant gsm1-1 and wild-type *Arabidopsis* [85]. Humphry et al. (2010) investigated the accumulation of indole GLS in several insertion lines, and the results suggested that MYB51 participates in the regulation of genes critical for GLS metabolism, which also influences antifungal defense [86]. Meanwhile, S-deficiency in oilseed rape can reduce GLS biosynthesis, which negatively affects resistance against *Leptosphaeria maculans*, *B. cinerea*, and *Phytophthora brassicae* [57].

According to Giamoustaris and Mithen (2010), the levels of *Alternaria* infection are positively correlated with napus GLS contents, and there is no significant relationship between the GLS content and *Leptosphaeria maculans* resistance [87]. In addition, Robin et al. (2020) found that GLS biosynthetic genes were induced following a study carried out on two resistant and two susceptible cabbage in-bred lines after inoculation with two *Leptosphaeria maculans* isolates, and GLS (aliphatic and indolic GLS) accumulation was enhanced [88]. In a study investigating the indolyl-3-acetonitrile, 4-methoxyglucobrassicin, and indole GLS concentrations in *B. rapa* inoculated with *Albugo candida*, Pedras et al. (2008) observed increased levels of indole GLS in inoculated leaves when compared to the control leaves [89]. *B. rapa* indole GLS has also been reported to limit *Colletotrichum gloeosporioides* and *Colletotrichum orbiculare* infection [90], and tryptophan pathway genes involved in indole-GLS biosynthesis are upregulated in *F. oxysporum*-infected plants [91,92].

Based on dynamic transcriptomic analyses of *B. rapus* defense response to *S. sclerotiorum* post-inoculation, Zhao et al. (2004), Borge et al. (2015), and Wu et al. (2016) observed that not only the GLS content but also indolic GLS biosynthesis are associated with *S. sclerotiorum* resistance, and that *S. sclerotiorum* infection can induce GLS biosynthesis [8,93,94]. Unlike in the case of *S. sclerotiorum*, *B. cinerea* does not induce GLS biosynthesis [95]. A comparison of the disease symptoms of wild-type and transgenic *Arabidopsis* lines following inoculating with arbuscular mycorrhizal fungi (AMF), based on the production or enhancement of GLS levels, revealed a previously undocumented role of GLS biosynthesis in reducing AMF colonization [96].

After *Plasmodiophora brassicae* infection, the aliphatic, indolic, and aromatic GLS contents of susceptible *B. napus* exhibit increased accumulation; however, only aromatic GLS contents are significantly increased in resistant *Matthiola incana* L. [97]. The major aliphatic GLS, gluconapin, is significantly increased during secondary infection in *B. napus*, and exogenous jasmonic acid (JA) treatment induces aliphatic GLS in *B. napus* and aromatic GLS in *M. incana*. The expression of *BnMYB28.1*, which regulates the contents of aliphatic GLS in *B. napus*, is significantly increased following both treatment with exogenous JA and *P. brassicae* inoculation. Similarly, after *B. cinerea* infection, the genes involved in indole GLS biosynthesis are upregulated in the *Arabidopsis* UGT80A2 and UGT80B1 double mutant, and the upregulation was correlated with increased levels of JA and the upregulation of two marker genes (PDF1.2 and PR4) of the ERF branch of the JA signaling pathway [98].

## 3. Defense Responses of GLS to Bacterial Diseases

The bacteria that infect cruciferous plants are all rod-shaped bacteria, which can invade the host through stomata, hydathodes, and wounds, and then be retransmitted by running water, rain, insects, etc. Bacterial diseases in cruciferous have widespread occurrence, are highly destructive, and are challenging to control. Meanwhile, because the pathogens are different from the fungal diseases, the corresponding disease resistance mechanism of host and the GLS involved in resistance may be different. Several studies have demonstrated that GLS are involved in plant defense against a variety of bacterial diseases. Similar to the case in fungal disease infection, infection by *Burkholderia cepacia*, *Pseudomonas syringae*, and *Xanthomonas campestris* pv. *campestris* (*Xcc*) led to the upregulation of the GLS biosynthesis [99]. In addition, the introduction of CYP79 influenced *Arabidopsis* disease resistance by increasing the GLS synthesis, and overexpressing the CYP79D2 from cassava increased the accumulation of the aliphatic isopropyl and methylpropyl GLS, which also enhanced resistance against the soft-rot pathogen, *Erwinia carotovora*; however, overexpressing the sorghum CYP79A1 or CYP79A2 increased the accumulation of p-hydroxybenzyl and benzyl GLS, respectively [100].

Mishina et al. (2007) observed that the knockout of PAL1 increased leaf survival after *P. syringae* infection in an analysis conducted on *Arabidopsis* mutants and wild type plants, while Truman et al. (2007) and Aires et al. (2011) observed that indole GLS biosynthesis decreased after *P. syringae* infection [101,102,103]. Following the transcriptional and metabolic profiling of *A. thaliana* mutants, Clay et al. (2009) reported that the PEN2 and PEN3 genes are necessary for resistance to PtoDC3000 pathogens [104]. Furthermore, Geng et al. (2012) demonstrated that coronatine, a toxin produced by *P. syringae*, suppresses the salicylic acid (SA)-independent pathway, facilitating callose deposition by reducing the accumulation of an indole GLS upstream of the PEN2 myrosinase activity [105]. In addition, a positive correlation has been reported between total GLS content and *Xcc* disease severity, and *Xcc* infection enhanced GLS biosynthesis during the early infection period [106,107]. *Pectobacterium carotovorum* ssp. *carotovorum* infection in *B. rapa* can trigger the upregulation of the JA and ethylene (ET) biosynthesis genes in sr gene mutants and increase resistance capacity via GLS accumulation [108].

## 4. Defense Response of GLS to Pests

With global warming, the loss caused by pests is increasing. Meanwhile, because pests have migration ability, once the control is not effective, it will cause serious damage [109]. Hence, pest control has been a hot spot in agriculture. At present, pest control is mainly focused on chemical agents, but how *Brassicaceae* plants perceive and defend themselves from such threats remain poorly understood. Investigating the mechanisms via which *Brassicaceae* resist insect pests could facilitate efforts to improve crop productivity. Brown and Morra (1997) were the first to report that GLS-containing plants could control soil-borne plant pests [110]. Since then, numerous studies have demonstrated that GLS contents in tissues are positively correlated with damage caused by *Pieris rapae* and *Spodoptera littoralis* [111,112], but negatively correlated with the damage caused by slugs [86]. Furthermore, GLS accumulation induced by *Spodoptera exigua* required functional NPR1 and ETR genes [113].

In another study, the weights of *Trichoplusiani* and *Manduca sexta* on the TGG1 and TGG2 double myrosinase mutants were significantly higher than in wild-type *Arabidopsis* [27]. Similarly, *Mamestra brassicae* larvae gained less weight and exhibited stunted growth when fed on MINELESS (lacking myrosin cells) plants compared to when fed on wild-type plants, with the myrosinase activity in the wild-type seedlings reducing; however, the levels of indol-3-yl-methyl, 1-methoxy-indol-3-yl-methyl, and total GLS in both the wild-type and MINELESS seedlings increased [114]. Conversely, *M. brassicae* and *P. rapae* weighed more on the high-sinigrin concentration plants than in low-sinigrin concentration plants; however, their weights decreased in the high-sinigrin, high-glucoiberin, and high-glucobrassicin genotypes; furthermore, development time increased under high glucobrassicin concentrations [115].

By testing the GLS and phenolic concentrations trends in *Brassica nigra* (L.) Koch before and after herbivory by *Pratylenchus penetrans* Cobb and the larvae *Delia radicum* L., Van et al. (2005) observed that the total GLS levels were affected by herbivory by the two root feeders [116]. Besides, *Spodoptera litura Fabricius* was more affected by induced GLS responses than *Plutella xylostella* L. [117]. In addition, following a comparison of GLS levels and the expression profiles of GLS biosynthesis genes before and after *Plutella xylostella* infestation, Liu et al. (2016) observed a difference in the proportions of stereoisomers of hydroxylated aromatic GLS between G-type (pest-resistant) and P-type (pest-susceptible) *Barbara vulgaris* [56]. Using *m*/*z* 60 as a marker of Al-ITC formation from the sinigrin GLS, Van et al. (2012) analyzed the GLS profiles and volatile organic compound emissions in five *Brassicaceae* species before and after artificial injury or infestation by cabbage root fly larvae (*D. radicum*). According to the results, *m*/*z* 60 in *B. nigra*, *B. juncea*, and *B. napus* was primarily emitted directly after artificial injury or root fly infestation, sulfide and methanethiol emissions from *B. nigra* and *B. juncea* increased after infestation, and *B. oleracea* and *Brassica carinata* exhibited increases in fig *m*/*z* 60 emissions following larval damage [118].

Long-term feeding on GLS-free *Brassicaceae* diets hardly affects *P. xylostella* oviposition preference and larvae survival; thus, high GLS content varieties are likely to be more susceptible to damage by *P. xylostella* than lower GLS content varieties [119]. Similarly, Chen et al. (2020) generated single or double mutant gss1 and gss2 lines using the CRISPR/Cas9 system and analyzed their resistance to *P. xylostella* [120]. According to the results of the bioassays, when fed on their usual artificial diet, there were significant reductions in egg hatching rates and final larval survival rate of the single mutant gss2 lines when compared with the original strain or mutant gss1 lines, and the absence of GSS1 or GSS2 reduced the survival rate of *P. xylostella* and prolonged the duration of the larval stage. In addition, feeding by *Spodoptera littoralis*, *Pieris brassicae*, and *P. rapae* led to upregulation of the aliphatic GLS pathway [121,122,123], and the GLS contents were negatively correlated with *P. brassicae* damage. Furthermore, methyl jasmonate (MeJA) can enhance resistance to *P. brassicae* by inducing GLS accumulation [124,125].

## 5. Defense Response of GLS to Insects and Aphids

Insects and aphid not only have a wide range of species and rapid reproduction, but also can cause wounds to the plant when feeding, leading to the invasion of pathogenic bacteria, and then cause secondary damage. Moreover, the GLS synthesis and response mechanisms following insect and aphid herbivory are qualitatively and quantitatively different [126]. Agerbirk et al. (2001) observed no correlation between *B. vulgaris* ssp. *arcuata* GLS content and resistance against *Phyllotreta nemorum* [127], while Kroymann et al. (2003) observed a positive correlation between GLS content and damage caused by *Psylliodes chrysocephala* [128]. According to Ulmer et al (2006), total GLS levels did not influence *Ceutorhynchus obstrictus* larval growth or development; however, high levels of specific GLS, such as p-hydroxybenzyl and 3-butenyl GLS, were associated with increased development time or reduced weight [129]. After *Brevicoryne brassicae* herbivory, Myrosinase binding protein (MBP), myrosinase associated protein (MyAP), and myrosinase transcripts, and the synthesis of indolyl and aliphatic GLS, particularly 3-hydroxypropyl and ITC, are induced [103,130,131].

By comparing the larval instar weights and mortality of cabbage stem flea beetle (*P. chrysocephala*) larvae, after feeding on different species, Döring et al. (2020) observed that aliphatic GLS contents increased in the infested turnip rape, and aliphatic and benzenic GLS decreased in infested Indian rape [132]. Although larval weight was not correlated with total GLS, it was positively correlated with progoitrin and 4-hydroxyglucobrassicin contents. Furthermore, decreasing the side chain length of aliphatic GLS and the degree of hydroxylation of butenyl GLS could increase the extent of feeding by adult flea beetles [87].

Numerous intermediate synthetic genes participate in GLS resistance to insects and aphids. For instance, Mewis et al. (2006) observed that GLS accumulation caused by *B. brassicae* and *Myzus persicae* required functional NPR1 and ETR1 genes [113]. After *Myzus persicae* feeding and aphid saliva treatment, a set of O-methyltransferases involved in the synthesis of aphid-repellent GLS were significantly up-regulated based on qRT-PCR analyses of 78 genes. However, ITC production was not correlated with these gene expression level, suggesting that aphid salivary components trigger a defense response in *Arabidopsis* that is independent of the aphid-deterrent GLS [133]. In addition, aphid attack could increase indolyl GLS concentrations three-fold [134]. Using a combination of QTL fine-mapping and microarray-based transcript profiling methods, CYP81F2 was revealed to facilitate defense against *B. brassicae* but not resistance against herbivory by larvae from four lepidopteran species [135]. By comparing the survival of the *Bemisia tabaci* MEAM1 and *B. tabaci* MED following exposure to sinigrin and myrosinase, Hu et al. (2020) reported that exposure to the toxic hydrolysates of GLS hydrolysates and myrosinase is greater for MED than for MEAM1 [136].

## 6. Conclusions and Future Research Outlook

Cruciferous vegetables are the most important leafy vegetables; however, the cultivation of cruciferous plants is affected by various fungi, bacteria, aphids, and other pest insects. At present, the prevention and control of these diseases and insect pests mainly focus on chemical agents, and the dosage of the chemical pesticides is also increasing, which not only leads to excessive pesticide residues on cruciferous plants, causing great damage to the environment, but also threatens people's health. Understanding how host-plant characteristics influence the physiological and behavioral responses is essential for the development of resistant cruciferous germplasms. A large number of studies have shown that GLS, esters, and flavonoids are closely related to Cruciferae disease resistance [137,138]. As an important secondary metabolite in cruciferous vegetables, GLS are closely related to biotic and abiotic stresses. Numerous studies have demonstrated a positive relationship between GLS content and disease and insect resistance [57,88,99,106,107,111,112,116,128,134] (Table 1). Consequently, in future cruciferous vegetable breeding activities, varieties with high GLS contents can be selected appropriately to improve plant disease resistance and reduce pesticide use. The degradation products (isothiocyanate and thiocyanate) of GLS are involved in the resistance to a variety of fungi, bacteria, insects, and soil-borne pests [8,71,72]; the aqueous extracts of cruciferous leaves also contain ITC, which can restrict the growth of a variety of fungi, bacteria, and pests [84,85,110]. Moreover, the resistance of these degradation products to pests and diseases is a broad-spectrum resistance, and thus can be used to develop botanical pesticides.

**Figure 3:**
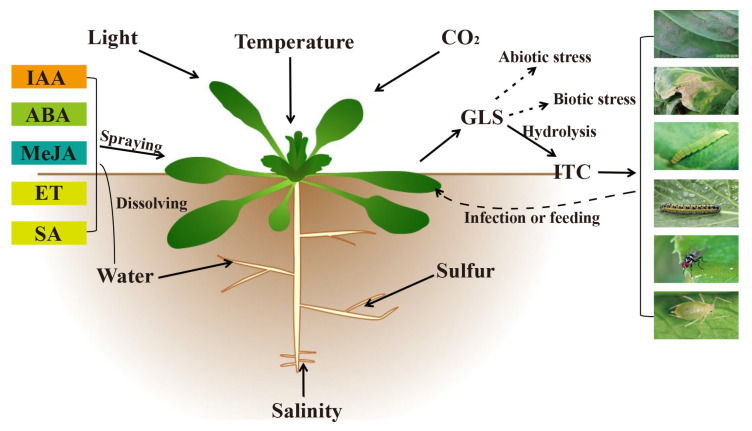
Factors affecting the synthesis of glucosinolate in cruciferous plants.

Pest invasion and disease infestation can increase GLS, especially indole GLS, in cruciferous plants [8,9,89,90,91,92,93,94,95,105,117,121,122,123,133,134]. In the case of rapeseed and other species that have low GLS, molecular biology techniques can be used to increase indole GLS production, which could improve resistance to diseases and insect, without increasing total GLS synthesis. Similar to other secondary metabolites, GLS synthesis is regulated by plant hormones. By controlling the amount of sulfur fertilizer applied and exogenous plant hormone treatments, such as JA, ET, MeJA, and SA, GLS synthesis can be modulated, and, in turn, disease resistance [57,105,125,126,139,140,141,142]. In addition, pathogen infection and insect herbivory can trigger the upregulation of the JA and ET biosynthesis genes, and increase defensive capacity via GLS accumulation [108]. Therefore, in subsequent cruciferous vegetable production activities, appropriate plant hormones could be sprayed as a novel pest management strategy to improve their stress resistance and minimize pesticide use (Figure 3).

Some studies have demonstrated that the different stereoisomer structures of hydroxylated aromatic GLS is one of the important factors influencing the varying disease resistance levels between non-cultivars and resistant cultivars [56]. Consequently, by determining and analyzing the GLS responsible for resistance in tolerant materials, chemical synthesis or biotechnology tools can be used to mass-produce the corresponding GLS for widespread application. The present review on the GLS responsible for disease and pest resistance in cruciferous vegetables and their underlying mechanisms could not only offers insights on how cruciferous plants could respond to increased biotic stress in the future but could also facilitate the development of novel disease and pest-resistant plants and the development of safe and high-yield cruciferous vegetable germplasms globally.

Despite there being many research studies on cruciferous plant resistance, which indicated the invasion of the diseases, insects and pests will lead to the synthetic form of the hormones and GLS, and total GLS, especially the indole GLS content, has a positive correlation with cruciferous resistance, but only a few studies have identified the specific resistance due to GLS and other metabolites. This may be related to the determination methods of GLS composition. At present, the conventional determination methods of GLS are HPLC (high performance liquid chromatography) and HPLC-MS; these two methods are not only expensive and difficult, but also often lack some standard samples. So, efficient and accurate GLS determination methods need to be improved or developed in subsequent research. Moreover, the synthesis mechanism and corresponding intermediate pathway of GLS are mainly focused on the model plant *A. thaliana*; studies on other species are thus few. The genomes and cultivation of most cruciferous vegetable patterns are different from *A. thaliana*. Compared with *Arabidopsis*, the genomes and cultivation patterns of Cruciferous plants are more complex. Although the genomes of many cruciferous plants have been sequenced, the genomes of the majority of species are still unknown, which has limited the study on the anabolism of GLS and other disease-resistance-related substances. With the reduction of the cost of genome sequencing, transcriptome sequencing, and omics analysis, it is believed that people will have a new understanding of the mechanism of GLS against diseases and insects.

## Figures and Tables

**Table 1:** Correlation of the GLS components and their metabolites in corresponding pathogen, pest, and insect resistance.

Component	Species	Names	Correlation	References
ITC; Allyl-ITC	Fungal	*Alternaria brassicicola*	positive	[84,85,86]
		*Plectosphaerella cucumerina*	positive	[86]
		*Botrytis cinerea*	positive	[86]
		*Fusarium oxysporum*	positive	[86]
		*Peronospora parasitica*	positive	[86]
Total GLS	Fungal	*Alternaria brassicicola*	positive	[92]
		*Leptosphaeria maculans*	No; positive	[92,93]
		*Sclerotinia sclerotiorum*	positive	[7,98,99]
		Arbuscular mycorrhizal fungi	positive	[101]
	Bacteria	*Burkholderia cepacia*	positive	[104]
		*Pseudomonas syringae*	positive	[104]
		*Xanthomonas campestris*	positive	[104,111,112]
		*Pectobacterium carotovorum*	positive	[113]
	Pest	*Pieris rapae*	positive	[114]
		*Spodoptera littoralis*	positive	[115]
		Slug	negative	[93]
		*Spodoptera exigua*	positive	[116]
		*Trichoplusia ni*	positive	[117]
		*Manduca sexta*	positive	[117]
		*Mamestra brassicae*	positive	[118]
		*Pratylenchus penetrans*	positive	[118]
		*Delia radicum* L.	positive	[118]
		*Spodoptera litura Fabricius*	positive	[121]
		*Plutella xylostella* L.	positive	[121,123,124]
		*Pieris brassicae*	positive	[128,129]
	Insect	*Phyllotreta nemorum*	No	[131]
		*Psylliodes chrysocephala*	positive	[132]
		*Ceutorhynchus obstrictus*	No	[133]
Indole GLS	Fungal	*Albugo candida*	positive	[94]
		*Colletotrichum gloeosporioides*	Positive	[95]
		*Colletotrichum orbiculare*	Positive	[95]
		*Fusarium oxysporum*	positive	[96,97]
		*Plasmodiophora brassicae*	positive	[102]
	Bacteria	*Pseudomonas syringae*	positive	[106,107,108]
Aliphatic GLS	Fungal	*Plasmodiophora brassicae*	positive	[102]
	Pest	*Spodoptera littoralis*	positive	[125]
		*Pieris brassicae*	positive	[125]
		*Pieris rapae*	positive	[126]
	Insect	*Psylliodes chrysocephala*	positive	[137]
Aromatic GLS	Fungal	*Plasmodiophora brassicae*	positive	[102]
	Pest	*Plutella xylostella* L.	positive	[60]
Benzenic GLS	Insect	*Psylliodes chrysocephala*	positive	[136]
Indolyl-3-acetonitrile, 4-methoxyglucobrassicin,	Fungal	*Albugo candida*	positive	[94]
Aliphatic isopropyl; methylpropyl GLS	Bacteria	*Erwinia carotovora*	positive	[105]
Indol-3-yl-methyl; 1-methoxy-indol-3-yl-methyl	Pest	*Mamestra brassicae*	positive	[118]
P-hydroxybenzyl; 3-butenyl	Insect	*Ceutorhynchus obstrictus*	positive	[133]
Sinigrin	Pest	*Pieris rapae*	negative	[119]
Glucobrassicin	Pest	*Pieris rapae*	positive	[119]

## References

[1] Ahmad M., Ali Q., Hafeez M.M., Malik A. (2021). Improvement for Biotic and Abiotic Stress Tolerance in Crop Plants. Biol. Clin. Sci. Res. J..

[2] Kliebenstein D.J., Osbourn A. (2012). Making New Molecules-evolution of Pathways for Novel Metabolites in Plants. Curr. Opin. Plant Biol..

[3] Rejeb I.B., Pastor V., Mauch-Mani B. (2014). Plant Responses to Simultaneous Biotic and Abiotic Stress: Molecular Mechanisms. Plants.

[4] Pastorczyk M., Bednarek P. (2016). The Function of Glucosinolates and Related Metabolites in Plant Innate Immunity. Adv. Bot. Res..

[5] Moller B.L. (2010). Functional Diversifications of Cyanogenic Glucosides. Curr. Opin. Plant Biol..

[6] Wu J., Baldwin I.T. (2010). New Insights into Plant Responses to the Attack from Insect Herbivores. Annu. Rev. Genet..

[7] Bouranis D.L., Malagoli M., Avice J.C., Bloem E. (2020). Advances in Plant Sulfur Research. Plants.

[8] Borges A., Abreu A.C., Ferreira C., Saavedra M.J., Simoes L.C., Simoes M. (2015). Antibacterial Activity and Mode of Action of Selected Glucosinolate Hydrolysis Products Against Bacterial Pathogens. J. Food Sci. Technol..

[9] Jeandroz S., Lamotte O. (2017). Editorial: Plant Responses to Biotic and Abiotic Stresses: Lessons from Cell Signaling. Front. Plant Sci..

[10] Gupta A., Hisano H., Hojo Y., Matsuura T., Ikeda Y., Mori I.C., Senthil-Kumar M. (2017). Global Profiling of Phytohormone Dynamics during Combined Drought and Pathogen Stress in Arabidopsis Thaliana Reveals ABA and JA as Major Regulators. Sci. Rep..

[11] Chisholm S.T., Coaker G., Day B., Staskawicz B.J. (2006). Host-microbe Interactions: Shaping the Evolution of the Plant Immune Response. Cell.

[12] Beran F., Kollner T.G., Gershenzon J., Tholl D. (2019). Chemical Convergence between Plants and Insects: Biosynthetic Origins and Functions of Common Secondary Metabolites. New Phytol..

[13] Ishida M., Hara M., Fukino N., Kakizaki T., Morimitsu Y. (2014). Glucosinolate Metabolism, Functionality and Breeding for the Improvement of Brassicaceae Vegetables. Breed Sci..

[14] Blazevic I., Montaut S., Burcul F., Olsen C.E., Burow M., Rollin P., Agerbirk N. (2020). Glucosinolate Structural Diversity, Identification, Chemical Synthesis and Metabolism in Plants. Phytochemistry.

[15] Fahey J.W., Zalcmann A.T., Talalay P. (2001). The Chemical Diversity and Distribution of Glucosinolates and Isothiocyanates among Plants. Phytochemistry.

[16] Halkier B.A., Gershenzon J. (2006). Biology and Biochemistry of Glucosinolates. Annu. Rev. Plant Biol..

[17] Clarke D.B. (2010). Glucosinolates, Structures and Analysis in Food. Anal. Methods.

[18] Agerbirk N., Olsen C.E. (2012). Glucosinolate Structures in Evolution. Phytochemistry.

[19] Sonderby I.E., Geu-Flores F., Halkier B.A. (2010). Biosynthesis of Glucosinolates—Gene Discovery and Beyond. Trends Plant Sci..

[20] Li Z., Liu Y., Li L., Fang Z., Yang L., Zhuang M., Zhang Y., Lv H. (2019). Transcriptome Reveals the Gene Expression Patterns of Sulforaphane Metabolism in Broccoli Florets. PLoS ONE.

[21] Andini S., Dekker P., Gruppen H., Araya-Cloutier C., Vincken J.P. (2019). Modulation of Glucosinolate Composition in Brassicaceae Seeds by Germination and Fungal Elicitation. J. Agric. Food Chem..

[22] Frerigmann H., Bottcher C., Baatout D., Gigolashvili T. (2012). Glucosinolates are Produced in Trichomes of Arabidopsis Thaliana. Front. Plant Sci..

[23] Hwang I.M., Park B., Dang Y.M., Kim S.Y., Seo H.Y. (2019). Simultaneous Direct Determination of 15 Glucosinolates in Eight Brassica Species by UHPLC-Q-Orbitrap-MS. Food Chem..

[24] Bekaert M., Edger P.P., Hudson C.M., Pires J.C., Conant G.C. (2012). Metabolic and Evolutionary Costs of Herbivory Defense: Systems Biology of Glucosinolate Synthesis. New Phytol..

[25] Kliebenstein D.J., Kroymann J., Mitchell-Olds T. (2005). The Glucosinolate-myrosinase System in an Ecological and Evolutionary Context. Curr. Opin. Plant Biol..

[26] Hopkins R.J., van Dam N.M., van Loon J.J. (2009). Role of Glucosinolates in Insect-plant Relationships and Multitrophic Interactions. Annu. Rev. Entomol..

[27] Barth C., Jander G. (2006). Arabidopsis Myrosinases TGG1 and TGG2 have Redundant Function in Glucosinolate Breakdown and Insect Defense. Plant J..

[28] Burow M., Halkier B.A. (2017). How does a Plant Orchestrate Defense in Time and Space? Using Glucosinolates in Arabidopsis as Case Study. Curr. Opin. Plant Biol..

[29] Koroleva O.A., Davies A., Deeken R., Thorpe M.R., Tomos A.D., Hedrich R. (2000). Identification of a New Glucosinolate-Rich Cell Type in Arabidopsis Flower Stalk. Plant Physiol..

[30] Husebye H., Chadchawan S., Winge P., Thangstad O.P., Bones A.M. (2002). Guard Cell- and Phloem Idioblast-specific Expression of Thioglucoside Glucohydrolase 1 (myrosinase) in Arabidopsis. Plant Physiol..

[31] Thangstad O.P., Gilde B., Chadchawan S., Seem M., Bones A.M. (2004). Cell Specific, Cross-species Expression of Myrosinases in Brassica Napus, Arabidopsis Thaliana and Nicotiana Tabacum. Plant Mol. Biol..

[32] van Dam N.M., Tytgat T.O.G., Kirkegaard J.A. (2008). Root and Shoot Glucosinolates: A Comparison of Their Diversity, Function and Interactions in Natural and Managed Ecosystems. Phytochem. Rev..

[33] Bjorkman M., Klingen I., Birch A.N., Bones A.M., Bruce T.J., Johansen T.J., Meadow R., Molmann J., Seljasen R., Smart L.E. (2011). Phytochemicals of Brassicaceae in Plant Protection and Human Health—Influences of Climate, Environment and Agronomic Practice. Phytochemistry.

[34] Blažević I., Radonić A., Mastelić J., Zekić M., Skočibušić M., Maravić A. (2010). Glucosinolates, Glycosidically Bound Volatiles and Antimicrobial Activity of Aurinia sinuata (Brassicaceae). Food Chem..

[35] Bones A.M., Rossiter J.T. (2006). The Enzymic and Chemically Induced Decomposition of Glucosinolates. Phytochemistry.

[36] Zhang Z., Ober J.A., Kliebenstein D.J. (2006). The Gene Controlling the Quantitative Trait Locus EPITHIOSPECIFIER MODIFIER1 Alters Glucosinolate Hydrolysis and Insect Resistance in Arabidopsis. Plant Cell.

[37] Burow M., Bergner A., Gershenzon J., Wittstock U. (2007). Glucosinolate Hydrolysis in Lepidium sativum—Identification of the Thiocyanate-forming Protein. Plant Mol. Biol..

[38] Esteve M. (2020). Mechanisms Underlying Biological Effects of Cruciferous Glucosinolate-Derived Isothiocyanates/Indoles: A Focus on Metabolic Syndrome. Front. Nutr..

[39] Bongoni R., Verkerk R., Steenbekkers B., Dekker M., Stieger M. (2014). Evaluation of Different Cooking Conditions on Broccoli (*Brassica oleracea* var. *italica*) to Improve the Nutritional Value and Consumer Acceptance. Plant Foods Hum. Nutr..

[40] Novotny C., Schulzova V., Krmela A., Hajslova J., Svobodova K., Koudela M. (2018). Ascorbic Acid and Glucosinolate Levels in New Czech Cabbage Cultivars: Effect of Production System and Fungal Infection. Molecules.

[41] Rhee J.H., Choi S., Lee J.E., Hur O.S., Assefa A.D. (2020). Glucosinolate Content in *Brassica* Genetic Resources and Their Distribution Pattern within and between Inner, Middle, and Outer Leaves. Plants.

[42] Keck A.S., Finley J.W. (2004). Cruciferous Vegetables: Cancer Protective Mechanisms of Glucosinolate Hydrolysis Products and Selenium. Integr. Cancer Ther..

[43] Dinkova-Kostova A.T., Kostov R.V. (2012). Glucosinolates and Isothiocyanates in Health and Disease. Trends Mol. Med..

[44] Lippmann D., Lehmann C., Florian S., Barknowitz G., Haack M., Mewis I., Wiesner M., Schreiner M., Glatt H., Brigelius-Flohe R. (2014). Glucosinolates from Pak Choi and Broccoli Induce Enzymes and Inhibit Inflammation and Colon Cancer Differently. Food Funct..

[45] Lee Y.R., Chen M., Lee J.D., Zhang J., Lin S.Y., Fu T.M., Chen H., Ishikawa T., Chiang S.Y., Katon J. (2019). Reactivation of PTEN Tumor Suppressor for Cancer Treatment through Inhibition of a MYC-WWP1 Inhibitory Pathway. Science.

[46] Liu B., Mao Q., Cao M., Xie L. (2012). Cruciferous Vegetables Intake and Risk of Prostate Cancer: A Meta-analysis. Int. J. Urol..

[47] Wu Q.J., Yang Y., Vogtmann E., Wang J., Han L.H., Li H.L., Xiang Y.B. (2013). Cruciferous Vegetables Intake and the Risk of Colorectal Cancer: A Meta-analysis of Observational Studies. Ann. Oncol..

[48] Liu X., Lv K. (2013). Cruciferous Vegetables Intake is Inversely Associated with Risk of Breast Cancer: A Meta-analysis. Breast.

[49] Lucarini E., Micheli L., Trallori E., Citi V., Martelli A., Testai L., de Nicola G.R., Iori R., Calderone V., Ghelardini C. (2018). Effect of Glucoraphanin and Sulforaphane against Chemotherapy-induced Neuropathic Pain: Kv7 Potassium Channels Modulation by H_2_S Release *In Vivo*. Phytother. Res..

[50] Abbaoui B., Lucas C.R., Riedl K.M., Clinton S.K., Mortazavi A. (2018). Cruciferous Vegetables, Isothiocyanates, and Bladder Cancer Prevention. Mol. Nutr. Food Res..

[51] Kellingray L., Le Gall G., Doleman J.F., Narbad A., Mithen R.F. (2020). Effects of In Vitro Metabolism of a Broccoli Leachate, Glucosinolates and S-methylcysteine Sulphoxide on the Human Faecal Microbiome. Eur. J. Nutr..

[52] Maina S., Misinzo G., Bakari G., Kim H.Y. (2020). Human, Animal and Plant Health Benefits of Glucosinolates and Strategies for Enhanced Bioactivity: A Systematic Review. Molecules.

[53] Dinkova-Kostova A.T., Talalay P. (2000). Persuasive Evidence that Quinone Reductase Type 1 (DT diaphorase) Protects Cells against the Toxicity of Electrophiles and Reactive Forms of Oxygen. Free Radic. Biol. Med..

[54] Rask L., Andréasson E., Ekbom B., Eriksson S., Bo P., Meijer J. (2000). Myrosinase: Gene Family Evolution and Herbivore Defense in Brassicaceae. Plant Mol. Biol..

[55] Kos M., Houshyani B., Wietsma R., Kabouw P., Vet L., Loon J., Dicke M. (2012). Effects of Glucosinolates on a Generalist and Specialist Leaf-chewing Herbivore and an Associated Parasitoid. Phytochemistry.

[56] Tongjin L., Xiaohui Z., Haohui Y., Niels A., Yang Q. (2016). Aromatic Glucosinolate Biosynthesis Pathway in Barbarea Vulgaris and Its Response to Plutella Xylostella Infestation. Front. Plant Sci..

[57] Dubuis P.-H., Marazzi C., Städler E., Mauch F. (2005). Sulphur Deficiency Causes a Reduction in Antimicrobial Potential and Leads to Increased Disease Susceptibility of Oilseed Rape. J. Phytopathol..

[58] Rausch T., Wachter A. (2005). Sulfur Metabolism: A Versatile Platform for Launching Defence Operations. Trends Plant Sci..

[59] Mocniak L.E., Elkin K., Bollinger J.M. (2020). Lifetimes of the Aglycone Substrates of Specifier Proteins, the Autonomous Iron Enzymes That Dictate the Products of the Glucosinolate-Myrosinase Defense System in Brassica Plants. Biochemistry.

[60] Santamaria M.E., Garcia A., Arnaiz A., Rosa-Diaz I., Romero-Hernandez G., Diaz I., Martinez M. (2021). Comparative Transcriptomics Reveals Hidden Issues in the Plant Response to Arthropod Herbivores. J. Integr. Plant Biol..

[61] Calmes B., N’Guyen G., Dumur J., Brisach C.A., Campion C., Iacomi B., Pigné S., Dias E., Macherel D., Guillemette T. (2015). Glucosinolate-derived Isothiocyanates Impact Mitochondrial Function in Fungal Cells and Elicit an Oxidative Stress Response Necessary for Growth Recovery. Front. Plant Sci..

[62] Pereira F., Rosa E., Fahey J.W., Stephenson K.K., Carvalho R., Aires A. (2002). Influence of Temperature and Ontogeny on the Levels of Glucosinolates in Broccoli (*Brassica oleracea* Var. *italica*) Sprouts and Their Effect on the Induction of Mammalian Phase 2 Enzymes. J. Agric. Food Chem..

[63] LoPez-Berenguer C., MartiNez-Ballesta M.D., Moreno D., Carvajal M., Garcia-Viguera C. (2009). Growing Hardier Crops for Better Health: Salinity Tolerance and the Nutritional Value of Broccoli. J. Agric. Food Chem..

[64] Yuan G., Wang X., Guo R., Wang Q. (2010). Effect of Salt Stress on Phenolic Compounds, Glucosinolates, Myrosinase and Antioxidant Activity in Radish Sprouts. Food Chem..

[65] Safavi Fard N., Heidari Sharif Abad H., Shirani Rad A.H., Majidi Heravan E., Daneshian J. (2018). Effect of Drought Stress on Qualitative Characteristics of Canola Cultivars in Winter Cultivation. Ind. Crop. Prod..

[66] Eom S.H., Baek S.A., Kim J.K., Hyun T.K. (2018). Transcriptome Analysis in Chinese Cabbage (*Brassica rapa* ssp. pekinensis) Provides the Role of Glucosinolate Metabolism in Response to Drought Stress. Molecules.

[67] Huseby S., Koprivova A., Lee B.R., Saha S., Mithen R., Wold A.B., Bengtsson G.B., Kopriva S. (2013). Diurnal and Light Regulation of Sulphur Assimilation and Glucosinolate Biosynthesis in *Arabidopsis*. J. Exp. Bot..

[68] del Carmen Martinez-Ballesta M., Moreno D.A., Carvajal M. (2013). The Physiological Importance of Glucosinolates on Plant Response to Abiotic Stress in *Brassica*. Int. J. Mol. Sci..

[69] Vale A.P., Santos J., Brito N.V., Fernandes D., Rosa E., Oliveira M.B. (2015). Evaluating the Impact of Sprouting Conditions on the Glucosinolate Content of *Brassica Oleracea* Sprouts. Phytochemistry.

[70] Xuan W., Beeckman T., Xu G. (2017). Plant Nitrogen nutrition: Sensing and Signaling. Curr. Opin. Plant Biol..

[71] Sanchez-Pujante P.J., Borja-Martinez M., Pedreno M.A., Almagro L. (2017). Biosynthesis and Bioactivity of Glucosinolates and Their Production in Plant In Vitro Cultures. Planta.

[72] Park S., Rim S.J., Jo M., Lee M.G., Kim C.E. (2019). Comorbidity of Alcohol Use and Other Psychiatric Disorders and Suicide Mortality: Data from the South Korean National Health Insurance Cohort, 2002 to 2013. Alcohol. Clin. Exp. Res..

[73] Korbas M., Jajor E., Budka A. (2009). Clubroot (*Plasmodiophora Brassicae*)—A Threat for Oilseed Rape. J. Plant Prot. Res..

[74] Yu F., Zhang W., Wang S., Wang H., Yu L., Zeng X., Fei Z., Li J. (2021). Genome Sequence of *Fusarium Oxysporum* f. sp. *Conglutinans*, the Etiological Agent of Cabbage Fusarium Wilt. Mol. Plant Microbe Interact..

[75] Jensen B., Hockenhull J., Munk L. (1999). Seedling and Adult Plant Resistance to Downy Mildew (*Peronospora parasitica*) in Cauliflower (*Brassica oleracea* convar. *botrytis* var. *botrytis*). Plant Pathol..

[76] Mahalingam T., Chen W., Rajapakse C.S., Somachandra K.P., Attanayake R.N. (2020). Genetic Diversity and Recombination in the Plant Pathogen *Sclerotinia Sclerotiorum* Detected in Sri Lanka. Pathogens.

[77] Samal B., Chatterjee S. (2019). New Insight into Bacterial Social Communication in Natural Host: Evidence for Interplay of Heterogeneous and Unison Quorum Response. PLoS Genet..

[78] Zhang S.-H., Yang Q., Ma R.-C. (2007). *Erwinia Carotovora* ssp. *Carotovora* Infection Induced “Defense Lignin” Accumulation and Lignin Biosynthetic Gene Expression in Chinese Cabbage (*Brassica rapa* L. ssp. *pekinensis*). J. Integr. Plant Biol..

[79] Takikawa Y., Takahashi F. (2014). Bacterial Leaf Spot and Blight of Crucifer Plants (Brassicaceae) Caused by *Pseudomonas Syringae* pv. *Maculicola* and *P. cannabina* pv. *alisalensis*. J. Gen. Plant Pathol..

[80] Gong J., Ju H.K., Kim I.H., Seo E.Y., Cho I.S., Hu W.X., Han J.Y., Kim J.K., Choi S.R., Lim Y.P. (2019). Sequence Variations Among 17 New Radish Isolates of *Turnip mosaic virus* Showing Differential Pathogenicity and Infectivity in *Nicotiana benthamiana*, *Brassica rapa*, and *Raphanus sativus*. Phytopathology.

[81] Iamba K., Malapa S. (2020). Efficacy of Selected Plant Extracts against Diamondback Moth (*Plutella xylostella* L.) on Round Cabbage In Situ. J. Entomol. Zool. Stud..

[82] Gabryś B., Pawluk M. (1999). Acceptability of Different Species of Brassicaceae as Hosts for the Cabbage Aphid. Proceedings of the 10th International Symposium on Insect-Plant Relationships.

[83] Griese E., Pineda A., Pashalidou F.G., Iradi E.P., Fatouros N.E. (2020). Plant Responses to Butterfly Oviposition Partly Explain Preference–Performance Relationships on Different Brassicaceous Species. Oecologia.

[84] Tierens M.J., Thomma B., Brouwer M., Schmidt J., Kistner K., Porzel A., Mauch-Mani B., Broekaert C. (2001). Study of the Role of Antimicrobial Glucosinolate-Derived Isothiocyanates in Resistance of Arabidopsis to Microbial Pathogens. Plant Physiol..

[85] Sellam A., Dongo A., Guillemette T., Hudhomme P., Simoneau P. (2007). Transcriptional Responses to Exposure to the Brassicaceous Defence Metabolites Camalexin and Allyl-isothiocyanate in the Necrotrophic Fungus *Alternaria Brassicicola*. Mol. Plant Pathol..

[86] Humphry M., Bednarek P., Kemmerling B., Koh S., Stein M., Gobel U., Stuber K., Pislewska-Bednarek M., Loraine A., Schulze-Lefert P. (2010). A Regulon Conserved in Monocot and Dicot Plants Defines a Functional Module in Antifungal Plant Immunity. Proc. Natl. Acad. Sci. USA.

[87] Giamoustaris A., Mithen R.F. (2010). Glucosinolates and Disease Resistance in Oilseed Rape (*Brassica napus* ssp. *oleifera*). Plant Pathol..

[88] Robin A.H.K., Laila R., Abuyusuf M., Park J.I., Nou I.S. (2020). Leptosphaeria Maculans Alters Glucosinolate Accumulation and Expression of Aliphatic and Indolic Glucosinolate Biosynthesis Genes in Blackleg Disease-Resistant and -Susceptible Cabbage Lines at the Seedling Stage. Front. Plant Sci..

[89] Pedras M.S., Zheng Q.A., Gadagi R.S., Rimmer S.R. (2008). Phytoalexins and Polar Metabolites from the Oilseeds Canola and Rapeseed: Differential Metabolic Responses to the Biotroph Albugo Candida and to Abiotic Stress. Phytochemistry.

[90] Hiruma K., Onozawa-Komori M., Takahashi F., Asakura M., Bednarek P., Okuno T., Schulze-Lefert P., Takano Y. (2010). Entry Mode-dependent Function of an Indole Glucosinolate Pathway in Arabidopsis for Nonhost Resistance against Anthracnose Pathogens. Plant Cell.

[91] Kidd B.N., Kadoo N.Y., Dombrecht B., Tekeoglu M., Kazan K. (2011). Auxin Signaling and Transport Promote Susceptibility to the Root-infecting Fungal Pathogen *Fusarium Oxysporum* in *Arabidopsis*. Mol. Plant Microbe Interact. MPMI.

[92] Zhu Q.H., Stephen S., Kazan K., Jin G., Fan L., Taylor J., Dennis E.S., Helliwell C.A., Wang M.B. (2013). Characterization of the Defense Transcriptome Responsive to *Fusarium Oxysporum*-infection in *Arabidopsis* Using RNA-seq. Gene.

[93] Zhao J., Peltier A.J., Meng J., Osborn T.C., Grau C.R. (2004). Evaluation of Sclerotinia Stem Rot Resistance in Oilseed *Brassica napus* Using a Petiole Inoculation Technique Under Greenhouse Conditions. Plant Dis..

[94] Wu J., Zhao Q., Yang Q., Liu H., Li Q., Yi X., Cheng Y., Guo L., Fan C., Zhou Y. (2016). Comparative Transcriptomic Analysis Uncovers the Complex Genetic Network for Resistance to *Sclerotinia Sclerotiorum* in *Brassica Napus*. Sci. Rep..

[95] Stotz H.U., Sawada Y., Shimada Y., Hirai M.Y., Sasaki E., Krischke M., Brown P.D., Saito K., Kamiya Y. (2011). Role of Camalexin, Indole Glucosinolates, and Side Chain Modification of Glucosinolate-derived Isothiocyanates in Defense of Arabidopsis against *Sclerotinia Sclerotiorum*. Plant J..

[96] Anthony M.A., Celenza J.L., Armstrong A., Frey S.D. (2020). Indolic Glucosinolate Pathway Provides Resistance to Mycorrhizal Fungal Colonization in a Non-host Brassicaceae. Ecosphere.

[97] Xu L., Yang H., Ren L., Chen W., Liu L., Liu F., Zeng L., Yan R., Chen K., Fang X. (2018). Jasmonic Acid-Mediated Aliphatic Glucosinolate Metabolism Is Involved in Clubroot Disease Development in *Brassica napus* L. Front. Plant Sci..

[98] Castillo N., Pastor V., Chavez A., Arro M., Boronat A., Flors V., Ferrer A., Altabella T. (2019). Inactivation of UDP-Glucose Sterol Glucosyltransferases Enhances *Arabidopsis* Resistance to *Botrytis cinerea*. Front. Plant Sci..

[99] Tinte M.M., Steenkamp P.A., Piater L.A., Dubery I.A. (2020). Lipopolysaccharide Perception in Arabidopsis Thaliana: Diverse LPS Chemotypes from Burkholderia Cepacia, Pseudomonas Syringae and Xanthomonas Campestris Trigger Differential Defence-related Perturbations in the Metabolome. Plant Physiol. Biochem..

[100] Brader G., Mikkelsen M.D., Halkier B.A., Tapio Palva E. (2006). Altering Glucosinolate Profiles Modulates Disease Resistance in Plants. Plant J..

[101] Mishina T.E., Zeier J. (2007). Bacterial Non-host Resistance: Interactions of *Arabidopsis* with Non-adapted *Pseudomonas Syringae* Strains. Physiol. Plant.

[102] Truman W., Bennettt M., Kubigsteltig I., Turnbull C., Grant M. (2007). Arabidopsis Systemic Immunity Uses Conserved Defense Signaling Pathways and is Mediated by Jasmonates. Proc. Natl. Acad. Sci. USA.

[103] Aires A., Dias C.S.P., Carvalho R., Oliveira M.H., Monteiro A.A., Simões M.V., Rosa E.A.S., Bennett R.N., Saavedra M.J. (2011). Correlations between Disease Severity, Glucosinolate Profiles and Total Phenolics and *Xanthomonas Campestris* pv. *Campestris* Inoculation of Different Brassicaceae. Sci. Hortic..

[104] Clay N.K., Adio A.M., Denoux C., Jander G., Ausubel F.M. (2009). Glucosinolate Metabolites Required for an *Arabidopsis* Innate Immune Response. Science.

[105] Geng X., Cheng J., Gangadharan A., Mackey D. (2012). The Coronatine Toxin of *Pseudomonas Syringae* is a Multifunctional Suppressor of *Arabidopsis* Defense. Plant Cell.

[106] Barah P., Winge P., Kusnierczyk A., Tran D.H., Bones A.M. (2013). Molecular Signatures in *Arabidopsis Thaliana* in Response to Insect Attack and Bacterial Infection. PLoS ONE.

[107] Sun Q., Zhang E., Liu Y., Xu Z., Hui M., Zhang X., Cai M. (2020). Transcriptome Analysis of Two Lines of *Brassica Oleracea* in Response to Early Infection with *Xanthomonas Campestris* pv. *Campestris*. Can. J. Plant Pathol..

[108] Liu M., Wu F., Wang S., Lu Y., Chen X., Wang Y., Gu A., Zhao J., Shen S. (2019). Comparative Transcriptome Analysis Reveals Defense Responses against Soft Rot in Chinese Cabbage. Hortic. Res..

[109] Deutsch C.A., Tewksbury J.J., Tigchelaar M., Battisti D.S., Merrill S.C., Huey R.B., Naylor R.L. (2018). Increase in Crop Losses to Insect Pests in a Warming Climate. Science.

[110] Brown P.D., Morra M.J. (1997). Control of Soil-Borne Plant Pests Using Glucosinolate-Containing Plants. Adv. Agron..

[111] Strauss S.Y., Lambrix I. (2004). Optimal Defence Theory and Flower Petal Colour Predict Variation in the Secondary Chemistry of Wild Radish. J. Ecol..

[112] Vadassery J., Reichelt M., Hause B., Gershenzon J., Boland W., Mithofer A. (2012). CML42-mediated Calcium Signaling Coordinates Responses to *Spodoptera* Herbivory and Abiotic Stresses in Arabidopsis. Plant Physiol..

[113] Mewis I., Appel H.M., Hom A., Raina R., Schultz J.C. (2005). Major Signaling Pathways Modulate Arabidopsis Glucosinolate Accumulation and Response to Both Phloem-feeding and Chewing Insects. Plant Physiol..

[114] Ahuja I., van Dam N.M., Winge P., Traelnes M., Heydarova A., Rohloff J., Langaas M., Bones A.M. (2015). Plant Defence Responses in Oilseed Rape *MINELESS* Plants after Attack by the Cabbage Moth *Mamestra Brassicae*. J. Exp. Bot..

[115] Santolamazza-Carbone S., Sotelo T., Velasco P., Cartea M.E. (2016). Antibiotic Properties of the Glucosinolates of *Brassica oleracea* var. *acephala* similarly Affect Generalist and Specialist Larvae of Two Lepidopteran Pests. J. Pest Sci..

[116] Dam N., Raaijmakers C.E., Putten W. (2005). Root Herbivory Reduces Growth and Survival of the Shoot Feeding Specialist *Pieris Rapae* on *Brassica Nigra*. Entomol. Exp. Appl..

[117] Mathur V., Ganta S., Raaijmakers C.E., Reddy A.S., Vet L.E.M., van Dam N.M. (2011). Temporal Dynamics of Herbivore-induced Responses in *Brassica Juncea* and Their Effect on Generalist and Specialist Herbivores. Entomol. Exp. Appl..

[118] van Dam N.M., Samudrala D., Harren F.J., Cristescu S.M. (2012). Real-time Analysis of Sulfur-containing Volatiles in *Brassica* Plants Infested with Root-feeding *Delia Radicum* Larvae Using Proton-transfer Reaction Mass Spectrometry. AoB Plants.

[119] Badenes-Pérez F.R., Gershenzon J., Heckel D.G. (2019). Plant Glucosinolate Content Increases Susceptibility to Diamondback Moth (Lepidoptera: Plutellidae) Regardless of Its Diet. J. Pest Sci..

[120] Chen W., Dong Y., Saqib H.S.A., Vasseur L., Zhou W., Zheng L., Lai Y., Ma X., Lin L., Xu X. (2020). Functions of Duplicated Glucosinolate Sulfatases in the Development and Host Adaptation of *Plutella Xylostella*. Insect Biochem. Mol. Biol..

[121] Ogran A., Faigenboim A., Barazani O. (2019). Transcriptome Responses to Different Herbivores Reveal Differences in Defense Strategies between Populations of *Eruca Sativa*. BMC Genom..

[122] Gols R., van Dam N.M., Reichelt M., Gershenzon J., Raaijmakers C.E., Bullock J.M., Harvey J.A. (2018). Seasonal and Herbivore-induced Dynamics of Foliar Glucosinolates in Wild Cabbage (*Brassica oleracea*). Chemoecology.

[123] Robert C.A.M., Pellissier L., Moreira X., Defossez E., Pfander M., Guyer A., van Dam N.M., Rasmann S. (2019). Correlated Induction of Phytohormones and Glucosinolates Shapes Insect Herbivore Resistance of Cardamine Species Along Elevational Gradients. J. Chem. Ecol..

[124] Buckley J., Pashalidou F.G., Fischer M.C., Widmer A., Mescher M.C., De Moraes C.M. (2019). Divergence in Glucosinolate Profiles between High- and Low-Elevation Populations of *Arabidopsis halleri* Correspond to Variation in Field Herbivory and Herbivore Behavioral Preferences. Int. J. Mol. Sci..

[125] Papazian S., Girdwood T., Wessels B.A., Poelman E.H., Dicke M., Moritz T., Albrectsen B.R. (2019). Leaf Metabolic Signatures Induced by Real and Simulated Herbivory in Black Mustard (*Brassica nigra*). Metabolomics.

[126] Kempema L.A., Cui X., Holzer F.M., Walling L.L. (2007). Arabidopsis Transcriptome Changes in Response to Phloem-Feeding Silverleaf Whitefly Nymphs. Similarities and Distinctions in Responses to Aphids. Plant Physiol..

[127] Agerbirk N., Olsen C.E., Nielsen J.K. (2001). Seasonal Variation in Leaf Glucosinolates and Insect Resistance in Two Types of *Barbarea Vulgaris* ssp. *arcuata*. Phytochemistry.

[128] Kroymann J., Donnerhacke S., Schnabelrauch D., Mitchell-Olds T. (2003). Evolutionary Dynamics of an *Arabidopsis* Insect Resistance Quantitative Trait Locus. Proc. Natl. Acad. Sci. USA.

[129] Ulmer B.J., Dosdall L.M. (2010). Glucosinolate Profile and Oviposition Behavior in Relation to the Susceptibilities of Brassicaceae to the Cabbage Seedpod Weevil. Entomol. Exp. Appl..

[130] Bo P., Hopkins R., Rask L., Meijer J. (2003). Infestation by Cabbage Aphid (*Brevicoryne brassicae*) on Oilseed Rape (*Brassica napus*) Causes a Long Lasting Induction of the Myrosinase System. Entomol. Exp. Appl..

[131] Kusnierczyk A., Winge P., Jorstad T.S., Troczynska J., Rossiter J.T., Bones A.M. (2008). Towards Global Understanding of Plant Defence against Aphids—Timing and Dynamics of Early Arabidopsis Defence Responses to Cabbage Aphid (*Brevicoryne brassicae*) Attack. Plant Cell Environ..

[132] Beukeboom L.W. (2020). Editor's Choice: March 2020. Entomol. Exp. Appl..

[133] de Vos M., Jander G. (2009). *Myzus Persicae* (green peach aphid) Salivary Components Induce Defence Responses in *Arabidopsis Thaliana*. Plant Cell Environ..

[134] Kuhlmann F., Muller C. (2009). Independent Responses to Ultraviolet Radiation and Herbivore Attack in Broccoli. J. Exp. Bot..

[135] Pfalz M., Vogel H., Kroymann J. (2009). The Gene Controlling the *Indole Glucosinolate Modifier1* Quantitative Trait Locus Alters Indole Glucosinolate Structures and Aphid Resistance in *Arabidopsis*. Plant Cell.

[136] Hu J., Yang J.J., Liu B.M., Cui H.Y., Zhang Y.J., Jiao X.G. (2020). Feeding Behavior Explains the Different Effects of Cabbage on MEAM1 and MED Cryptic Species of *Bemisia Tabaci*. Insect Sci..

[137] Anyanga M.O., Farman D.I., Ssemakula G.N., Mwanga R.O.M., Stevenson P.C. (2020). Effects of Hydroxycinnamic Acid Esters on Sweetpotato Weevil Feeding and Oviposition and Interactions with *Bacillus Thuringiensis* Proteins. J. Pest Sci..

[138] Yao Q., Peng Z., Tong H., Yang F., Xing G., Wang L., Zheng J., Zhang Y., Su Q. (2019). Tomato Plant Flavonoids Increase Whitefly Resistance and Reduce Spread of Tomato yellow leaf curl virus. J. Econ. Entomol..

[139] Jost R., Altschmied L., Bloem E., Bogs J., Gershenzon J., Hahnel U., Hansch R., Hartmann T., Kopriva S., Kruse C. (2005). Expression Profiling of Metabolic Genes in Response to Methyl Jasmonate Reveals Regulation of Genes of Primary and Secondary Sulfur-related Pathways in *Arabidopsis Thaliana*. Photosynth. Res..

[140] Pangesti N., Reichelt M., van de Mortel J.E., Kapsomenou E., Gershenzon J., van Loon J.J., Dicke M., Pineda A. (2016). Jasmonic Acid and Ethylene Signaling Pathways Regulate Glucosinolate Levels in Plants During Rhizobacteria-Induced Systemic Resistance Against a Leaf-Chewing Herbivore. J. Chem. Ecol..

[141] Kong W., Li J., Yu Q., Cang W., Xu R., Wang Y., Ji W. (2016). Two Novel Flavin-Containing Monooxygenases Involved in Biosynthesis of Aliphatic Glucosinolates. Front. Plant Sci..

[142] Lu C., Han M.H., Guevara-Garcia A., Fedoroff N.V. (2002). Mitogen-activated protein kinase signaling in postgermination arrest of development by abscisic acid. Proc. Natl. Acad. Sci. USA.

